# The Hospital Needs of Swindon

**Published:** 1923-10

**Authors:** 


### THE HOSPITAL NEEDS OF SWINDON.

### MR. ORDE'S REPORT.

A REPORT of great interest and importance has been issued by Mr. Roden Orde of the British Red Cross Society, on the hospital needs of Swindon. For some time the members of the Great Western Railway Medical Fund Society have been contemplating building another hospital for Swindon, and various sites have been mentioned, including the Great Western Railway Park. Mr. Orde recently spent four or five days in Swindon and went thoroughly into the question.

### A Grave Situation.

In a letter prefacing the report he writes :—

The Hospital situation at Swindon is a grave one, and calls for immediate consideration. How, or by what body action should first be taken, it is for those on the spot to determine. It would, however, appear not unreasonable to suggest that as a preliminary step the Mayor of the town might be invited to convene a meeting of representatives of the various bodies who are engaged in hospital and allied work (regional and local), of the medical profession generally, and of those citizens who are interested in public affairs. To such a gathering the situation and the suggested methods of meeting it might be clearly explained and a joint committee formed to consider and to report. On the report of such a committee future action would, of course, depend.

### The Victoria Hospital.

The total population of Swindon is 90,000, and of these 40,000 are employees of the Great Western Railway Company, with their wives and children. To serve this population are only forty-four beds :—

It will help to an understanding of the position to give a brief description of the chief health organisations that exist at Swindon, with their chief features. The Victoria Hospital was built in 1887 and contains thirty-four beds, entirely for surgical patients. This hospital may be described as a cottage hospital that has been suddenly called upon to perform the functions of a general hospital for a large population, and that has not yet had time to adjust its organisation, its buildings, and its finances to the task. The Victoria Hospital stands well on the high ground that in many respects is admirable for hospital purposes. Unfortunately the area available is not more than 1¼ acres. While the erection of a hospital of 150 beds, or thereabouts, on the present site is probably a building possibility, yet to do so would cover the ground and, in view of the fact that a portion of the present building would not carry an additional storey, leave little room for expansion. This crowding of the site is the less objectionable because the situation of the hospital is airy, the view to the south is mainly to open country, the houses on the west and east comparatively low, and the district generally a good residential one. What the possibilities of the present Victoria site may be have not yet been definitely ascertained. So far as our information goes, we understand that space to the south and east might possibly be obtained, and either new buildings erected on it or the houses already there utilised for hospital purposes.

### The Great Western Railway Hospital.

The Great Western Railway Medical Fund Society, founded in 1847, is an organisation confining its activities to the employees of the railway and their families :—

For these purposes a whole-time paid staff of ten surgeons and physicians are employed, three dental surgeons, seven dispensers, five nurses. The buildings give accommodation for twelve hospital beds, an operating theatre, waiting-hall, consulting-rooms, dressing-rooms, rooms for dental treatment (a very complete organisation with skilled workmen for the provision of artificial teeth, etc., the members being provided with sets at almost cost price), doctors' rooms, a large dispensary, swimming, Turkish and plunge baths, which the members can use at reduced fees, arrangements for funeral purposes, motor facilities for visiting doctors, etc. The whole of the buildings are close to the works. A portion of the main block is let off to the Corporation for maternity home purposes, and here there are eleven beds (a possible thirteen if required). With regard to its efficiency, I am bound to say I have been very greatly impressed.

### Three Suggestions.

To meet the generally acknowledged scarcity of beds in the town three suggestions have been made :—

1. To build on a new and open site on the outskirts of the town a hospital of 150 beds. 2. To extend the Victoria Hospital so as to provide 150 beds. 3. To build a hospital of 50 beds for the use of the employees of the Great Western Railway Company and their families at Swindon. Nos. 1 and 2 have not, so far as I understand, advanced beyond the suggestion stage. No. 3 has, however, assumed a definite shape and secured a certain amount of approval. The first suggestion that a general hospital of 150 beds should be built on an open site on the outskirts of the town is possibly the ideal one, and in the absence of any compromising factors it might have been justifiable to advise its adoption. The situation at Swindon, however, is compromised. A hospital on a good but limited site exists. Moreover, a sum of £19,000 has recently been spent on its extension.

### One Hospital for Swindon.

As regards the third suggestion Mr. Orde is inclined to think that the proposed site—ten acres laid out as a park and situated close to the railway—is not the best available, his reason being that he sees no preponderating advantage in the hospital being close to the railway works. Mr. Orde feels that the medical and surgical needs of the Swindon area would be best met by the establishment of one hospital for the whole population :—

So strongly do I hold this view that I would urge that no scheme should be proceeded with until the inhabitants as a whole have had the situation clearly explained to them, and with full knowledge have decided for or against joint action. Such consideration of so important a matter would in no way prejudice the freedom of the employees of the Great Western Railway Company, nor would it materially delay their project of building their own hospital should they determine to carry it out; but it is impossible to avoid the conclusion that this project owes its origin more to anxiety to get something done, than to any disinclination to take part in a movement for the general good.

### Joint Action Recommended.

The report is definitely in favour of joint action and the establishment of a hospital on the Victoria site :—

I would suggest that, should joint action be decided upon and the Victoria site be approved, plans should be at once prepared for the erection of a hospital of 150 beds, and I would further suggest that a portion of this extension should be put in hand with as little delay as possible—viz., the addition of 50 beds to the existing 38. Eighty-eight beds would give breathing space and relieve the situation almost to the extent contemplated by the erection of a separate hospital by the Medical Fund. It is, I think, essential not only to relieve the present natural anxiety of the employees of the Swindon Railway Works, but also to give some earnest of complete relief in the reasonably near future. Should it eventually and unfortunately prove impossible to erect a satisfactory hospital on the Victorian site owing to lack of space, then I am of opinion that attention should be turned to the building of a new hospital on a new site, so firmly am I persuaded that the interests of Swindon generally would be best served by one hospital of 150 to 200 beds, and not by two of smaller size.

